# Reprogramming mediated radio-resistance of 3D-grown cancer cells

**DOI:** 10.1093/jrr/rrv018

**Published:** 2015-04-16

**Authors:** Gang Xue, Zhenxin Ren, Peter W. Grabham, Yaxiong Chen, Jiayun Zhu, Yarong Du, Dong Pan, Xiaoman Li, Burong Hu

**Affiliations:** 1 Department of Space Radiobiology, Key Laboratory of Heavy Ion Radiation Biology and Medicine, Institute of Modern Physics, Chinese Academy of Sciences, 509 Nanchang Road, Building 5–204, Lanzhou 730000, China; 2 University of Chinese Academy of Sciences, Beijing, 100049, China; 3 Center for Radiological Research, College of Physicians and Surgeons, Columbia University, New York, 10032

**Keywords:** 3D growth microenvironment, matrigel, reprogramming, β-catenin, radio-resistance

## Abstract

*In vitro* 3D growth of tumors is a new cell culture model that more closely mimics the features of the *in vivo* environment and is being used increasingly in the field of biological and medical research. It has been demonstrated that cancer cells cultured in 3D matrices are more radio-resistant compared with cells in monolayers. However, the mechanisms causing this difference remain unclear. Here we show that cancer cells cultured in a 3D microenvironment demonstrated an increase in cells with stem cell properties. This was confirmed by the finding that cells in 3D cultures upregulated the gene and protein expression of the stem cell reprogramming factors such as OCT4, SOX2, NANOG, LIN28 and miR-302a, compared with cells in monolayers. Moreover, the expression of β-catenin, a regulating molecule of reprogramming factors, also increased in 3D-grown cancer cells. These findings suggest that cancer cells were reprogrammed to become stem cell–like cancer cells in a 3D growth culture microenvironment. Since cancer stem cell–like cells demonstrate an increased radio-resistance and chemo-resistance, our results offer a new perspective as to why. Our findings shed new light on understanding the features of the 3D growth cell model and its application in basic research into clinical radiotherapy and medicine.

## INTRODUCTION

Traditional 2D monolayer cell cultures are commonly used in current biological and medicinal studies. However, significant differences between the 2D cell culture system and an actual physiological environment *in vivo* have long been realized. First, cells *in vivo* are 3D and exhibit a round morphology due to a tightly controlled interplay between the cell and its extracellular matrix (ECM) focal adhesions and actin cytoskeleton [1]. Second, cells *in vivo* interact with the environment in a 3D manner. They are subjected to mechanical forces from the ECM and soluble chemicals. In contrast, when grown in traditional culture, such as 2D flat tissue culture substrates, cells do not simulate the structural organization of 3D tissues and, therefore, differ considerably in their morphology and cell–cell and cell–matrix interactions [2–4]. As a result, these 2D monolayer cells can't recapitulate the physiological conditions of *in vivo* microenvironments. As animal models and *in vivo* studies are costly and complex, with problems of unpredictable characteristics and ethical approval, physiological 3D model systems using human cells to create an authentic model is an obvious choice [5]. 3D cell culture is a third model bridging the gap between traditional cell culture and animal models [6, 7].

Matrigel basement membrane matrix (BD Biosciences) is a commercial cell culture medium comprised of a gelatinous protein mixture secreted by Engelbreth–Holm–Swarm (EHS) mouse sarcoma cells. It is rich in ECM components and was used widely for 3D cell culture. Cells cultured in matrigel show many differences in gene and protein expression, survival, proliferation, differentiation and metabolism when compared with traditional 2D culture cells [8–10]. In addition, the response behaviors of cells in 2D cultures and 3D cultures also differ [11, 12]. It has been demonstrated that 3D-cultured cancer cells are more radio-resistant and chemo-resistant compared with 2D monolayers; specifically, they show increased clonogenicity and resistance to apoptosis [13–15]. However, the reason behind the difference in radio-resistance and chemo-resistance between 2D- and 3D-grown cancer cells remains largely unknown. As is well known, matrigel is reported to help in maintaining a stem cell phenotype and in controlling the differentiation of stem cells [16], but the effect of matrigel on cancer cell reprogramming remains unknown. Thus we speculated whether the 3D growth microenvironment might have some impact on the reprogramming of differentiated cancer cells and in turn enhance the radio-resistance.

To test our hypothesis, we cultured A549 cancer cells in a 3D matrigel microenvironment. Our results showed that reprogramming factors such as OCT4, SOX2, NANOG, LIN28 and miR-302a were upregulated significantly in 3D-cultured cancer cells compared with their monolayer counterparts. 3D-cultured cancer cells were reprogrammed and acquired stem cell-like properties, and in turn demonstrated enhanced radio-resistance.

## MATERIALS AND METHODS

### Cell culture

A549 cells (adenocarcinomic human alveolar basal epithelial cells), MCF7 cells (human breast cancer cells) and PC3 cells (human prostate cancer cells) were obtained from the American Type Culture Collection (Manassas, VA, USA). For 2D-grown cultures, A549 cells were cultured in RPMI-1640 medium (Gibco, USA) supplemented with 10% FBS (Hyclone, USA) and 1% penicillin/streptomycin (Amresco, USA). MCF7 cells and PC3 cells were cultured in Dulbecco' Modified Eagle's Medium (DMEM) (Gibco, USA) supplemented with 10% FBS and 1% penicillin/streptomycin. For 3D-grown cultures, construction of the 3D growth microenvironment using matrigel (BD, USA) was performed mainly as described previously [17]. Briefly, a pre-chilled culture surface was coated with a thin layer of medium-matrigel mixture (volume ratio 1:1) and incubated for 30 min at 37°C to allow the mixture to gel. We then trypsinized 2D-cultured cells and mixed them at a concentration of 0.5 × 10^6^ cells/ml with matrigel (volume ratio 1:1). This was pipetted onto the pre-coated surface and incubated for 30 min at 37°C to allow them to gel. All experiments with 3D-grown cells were cultured in matrigel for 24 h. Both 2D- and 3D-grown cells were cultured at 37°C in a humidified atmosphere containing 5% CO_2_.

### Radiation

X-ray irradiation was carried out by a Faxitron RX-650 facility (Faxitron Bioptics, USA), which was operated at 50 kVp 5 mA at room temperature. The target of this instrument is wolframium (W). The dose rate was 0.751 Gy/min.

### Colony formation assay

For 2D culture, cells were trypsinized after radiation and resuspended in medium. An appropriate number of cells were plated into each 60-mm dish to produce colonies. For 3D culture, the irradiated and control cells were first recovered from matrigel by using Recovery Solution (BD, USA) on ice for 30 min, and then trypsinized and resuspended in medium. An appropriate number of cells were plated into each 60-mm dish to produce colonies. After incubating for 10 days, both 2D- and 3D-grown cells were stained with 0.5% crystal violet for 20 min. Colonies containing >50 cells were counted as survivors. Plating efficiencies (PE) were calculated as follows: numbers of colonies formed/numbers of cells plated. Surviving fractions were calculated as follows: PE (irradiated)/PE (unirradiated).

### Rhodamine staining analysis

2D- and 3D-cultured cells were harvested and prepared as single-cell suspensions in medium. The cells were stained with 5 μg/ml Rhodamine 123 (Invitrogen, USA) and incubated in darkness at 37°C for 10 minutes. After staining, the cells were washed with medium twice and incubated again in darkness with medium at 37°C for 60 min. The Rho fluorescence of stained cells was assayed using a flow cytometer (MACS, Germany).

### QRT-PCR for mRNA expression

Total RNAs of 2D- and 3D-cultured cells were extracted with TRIzol Reagent (Life Technologies, USA). For mRNA detection, reverse transcription was carried out with Transcriptor First Strand cDNA Synthesis Kit (Roche, Switzerland), and qRT-PCR was carried out with SYBR Green PCR Master (Roche, Switzerland). The primers of OCT4, SOX2, NANOG, c-MYC, LIN28 and internal control of GAPDH were purchased from GeneCopoeia (Guangzhou, China). For miRNA detection, reverse transcription and qRT-PCR was performed by using an ALL-in-one^TM^ miRNA qRT-PCR Detection Kit (GeneCopoeia, Guangzhou, China). The primers of miR-302a and internal control U6 were also purchased from GeneCopoeia. Q-PCR was performed on samples using a Bio-Rad Chromo4 System Real-Time PCR detector (Bio-Rad, USA). All steps were carried out according to the protocol. The relative fold change of mRNA was calculated by using the 2^−ΔΔCt^ method.

### Western blot

2D- and 3D-grown cells were lysed in RIPA buffer (Beyotime, Shanghai, China) with Protease Inhibitor Cocktail Tablets (Roche, Switzerland). The total protein concentrations of the lysates were determined using a protein assay kit (Bio-Rad, USA). Equal amounts of protein were denatured with loading buffer (Beyotime, Shanghai, China) at 100°C for 10 min, then loaded in 12% SDS-PAGE for electrophoresis, and transferred to a methanol-activated polyvinylidene fluoride membrane (Millipore, USA). The membrane was blocked in tris-buffered saline (TBS) containing 5% bovine serum albumin (MP Biomedical, USA) for 2 h at room temperature. Primary antibodies were incubated overnight at 4°C. The primary antibodies included: OCT4 (1:1000, Abcam, USA), SOX2 (1:1000, CST, USA), NANOG (1:1000, ABGENT, USA), β-catenin (1:1000, CST, USA) and GAPDH (1:1000, ZSGB-BIO, Beijing, China). After washing with TBS, the membrane was incubated with the appropriate horseradish peroxidase (HRP)- labeled secondary antibody for 1 h at room temperature. Secondary antibodies conjugated with HRP include: Goat-Anti Rabbit IgG (1:5000, ZSGB-BIO, Beijing, China) and Rabbit-Anti Goat IgG (1:5000, ZSGB-BIO, Beijing, China).

### Statistical analysis

The statistical significance (*P* value) for the mean value two-sample comparison was determined with Students' *t*-test. A value of *P* < 0.05 was considered statistically significant and is represented by an asterisk on the bars in the figures. A value of *P* < 0.01 was considered extremely significant and is represented by two asterisks on the bars in the figures. Values shown on graphs represent the means ± s.d.

## RESULTS

### Different morphology and radio-resistance of 3D-cultured cells compared with 2D-cultured cells

**Fig. 1.**
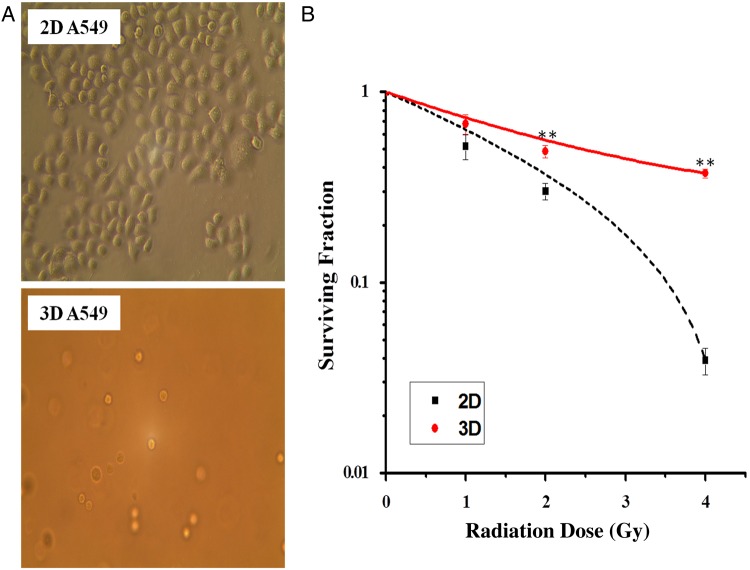
The morphology and clonogenic survival of 2D- and 3D-grown A549 cells. (**A**) Phase-contrast images of the 2D monolayer (above) and the 3D-cultured (below) A549 cells. (**B**) Survival fraction of 2D- and 3D-cultured A549 cells after exposure to 0, 1, 2 or 4 Gy of X-rays . Data are presented as mean ± SE. Experiments were independently repeated at least three times.

As shown in Fig. 1A, there was a change in cell shape between 2D-cultured and 3D-cultured cells. 2D-grown cells grew flat and spread as a monolayer on the tissue-culture dish, while 3D-grown cells cultured within the matrigel microenvironment showed a round morphology. 2D- and 3D-grown A549 cells differ in cell morphology, and 3D culture systems more closely approximate the cells' normal physiological environment *in vivo.* It has been demonstrated that 3D-grown cells are radio-resistant and chemo-resistant compared with cells grown in monolayers. Here we chose radiation as a stress method to test whether A549 cells cultured 3D in a microenvironment have increased radio-resistance. As shown (Fig. 1B), the clonogenic survival fraction of the 3D-grown A549 cells was significantly higher than that of the 2D-cultured cells after irradiation with X-rays.

### Increasing population of Rho-low–staining (Rho^low^) cells in 3D-cultured cells compared with 2D-cultured cells

**Fig. 2.**
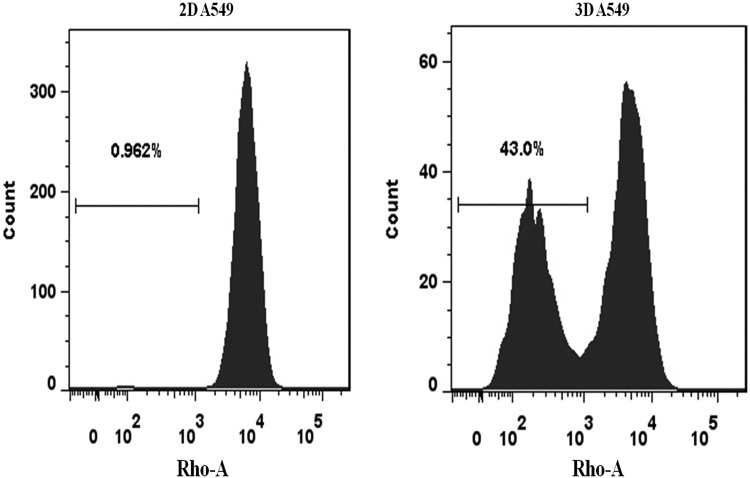
The percentage of stem cell–like cells in 2D- (left) and 3D- (right) cultured A549 cells assayed using Rho/FACS; the percentage of Rho^low^ cells is shown in the picture.

Hoechst is used to distinguish stem and non-stem cells, due to the fact that it can be excluded from cells by the ABC transporter, a specific protein found in stem cells [18]. Rhodamine 123 (Rho), another fluorescent dye, has also been used to identify stem cell–like cells by Rho/FACS [19, 20]. We determined the stem cell–like cell percentage in 2D- and 3D-grown A549 cells using the Rho/FACS method. As shown in Fig. 2, the percentage of Rho^low^ cells in 2D-grown A549 cells was 0.962%, whereas in 3D-grown cells the percentage of Rho^low^ cells was 43%. This result indicates that there was a significantly increased population of stem cell–like cells in 3D-cultured A549 cells.

### Upregulation of reprogramming factors in 3D-cultured cancer cells

**Fig. 3.**
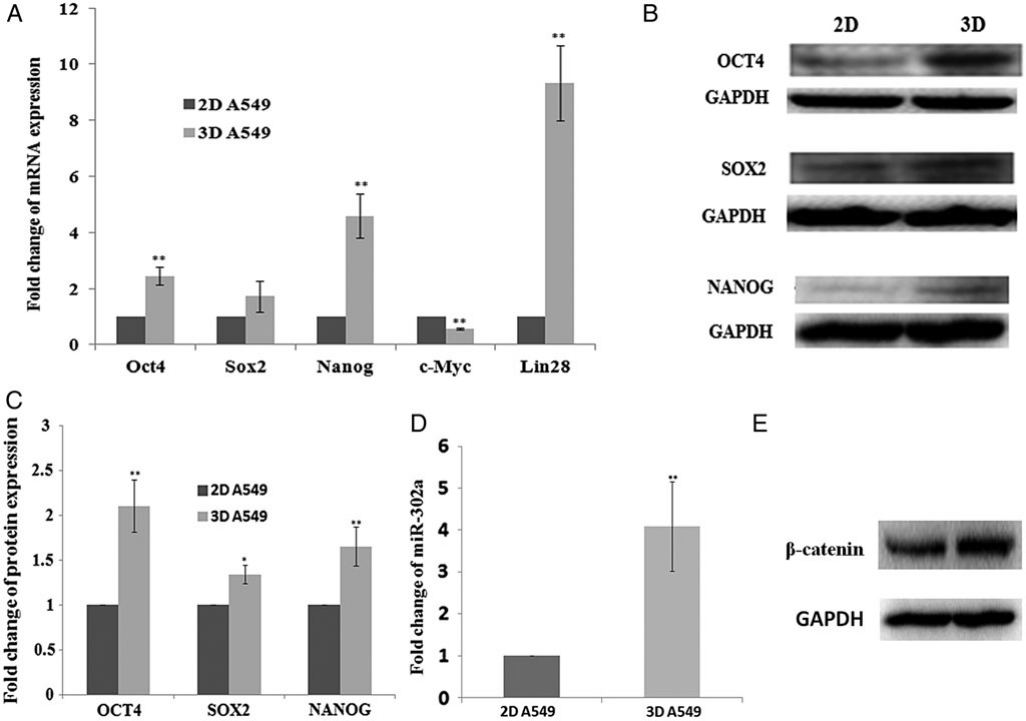
The expression of reprogramming transcription factors in 2D- and 3D-grown A549 cells. (**A**) qRT-PCR for the expression of reprogramming transcription factors OCT4, SOX2, NANOG, c-MYC and LIN28 in 2D- and 3D-grown A549 cells. (**B**) Western blotting for expression of OCT4, SOX2 and NANOG in 2D- and 3D-grown A549 cells. (**C**) Gray analysis the result of Figure 3B. (**D**) qRT-PCR for the expression of reprogramming factor miR-302a expression in 2D- and 3D-grown A549 cells. (**E**) Western blotting for the expression of β-catenin in 2D- and 3D-grown A549 cells. Data are presented as mean ± SE. Experiments were independently repeated at least three times.

To further investigate whether cancer cells cultured in a 3D matrigel microenvironment acquire stem cell–like characteristics when compared with 2D monolayer cells, we measured the expressions of the stem cell markers OCT4, SOX2, NANOG, c-MYC and LIN28 in both 2D- and 3D-cultured A549 cells. We first analyzed the mRNA expression levels of OCT4, SOX2, NANOG, c-MYC and LIN28 in 2D- and 3D-cultured A549 cells by qRT-PCR. As shown in Fig. 3A, we found that compared with 2D monolayer cells, the expressions of OCT4, NANOG and LIN28 increased in 3D-cultured cells, while the expression of c-MYC decreased in 3D-cultured cells. Further, we analyzed the protein expression levels of OCT4, SOX2 and NANOG in 2D- and 3D-grown A549 cells by western blot. We found that the protein levels of OCT4, SOX2 and NANOG were significantly upregulated in 3D-grown cells (Fig. 3B and 3C). Moreover, we measured the expression of miR-302a in both 2D- and 3D-grown A549 cells. We found that the expression of miR-302a was also upregulated in 3D-grown A549 cells (Fig. 3D). Earlier studies have demonstrated that β-catenin, a key downstream component of the canonical Wnt signaling pathway, can activate OCT4, LIN28, NANOG and miR-302 expression [21–24]. We therefore measured the expression of β-catenin in 2D- and 3D-grown cells. As shown in Fig. 3E, there was a significantly increased expression of β-catenin in 3D-grown A549 cells compared with monolayer cells. These results suggested that the 3D culture microenvironment induced the cancer cells to express reprogramming factors.

### More Rho^low^-staining cells in other 3D-grown cancer cells

**Fig. 4.**
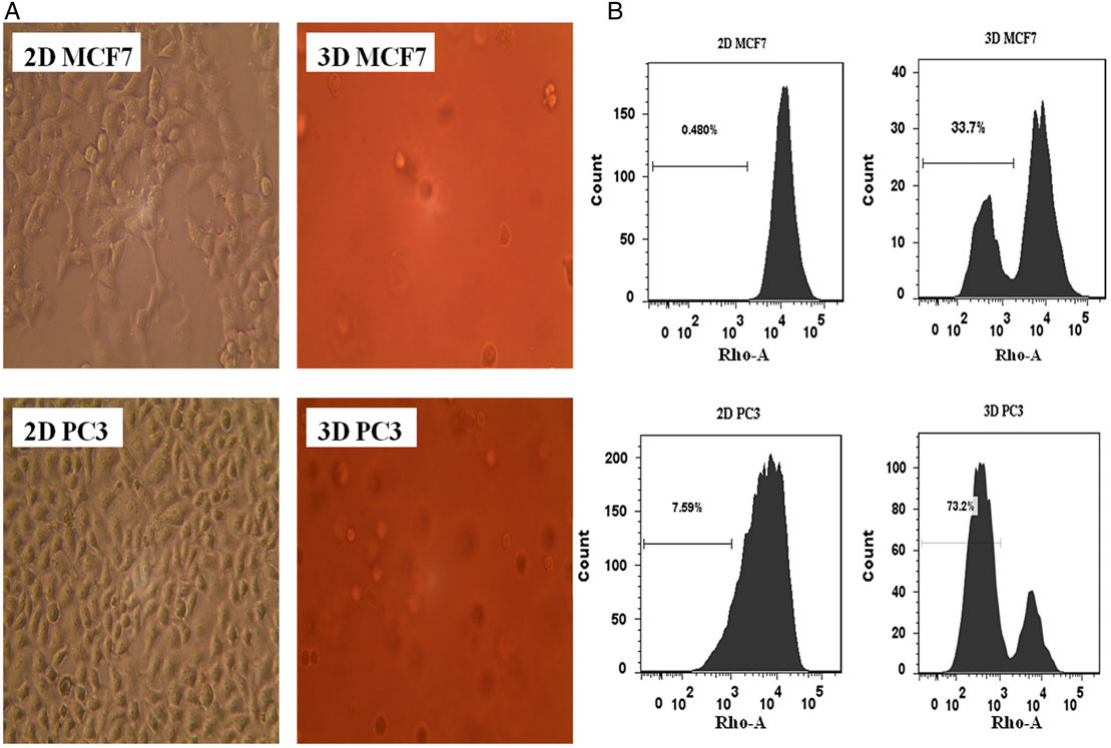
The morphology and stem cell-like phenotype of MCF7 and PC3 cancer cells in a 3D microenvironment, compared with 2D monolayer cells. (**A**) Phase-contrast images of the 2D- (left) and 3D- (right) grown MCF7 (above) and PC3 (below) cells. (**B**) The percentage of stem cell–like cells in 2D- (left) and 3D- (right) grown MCF7 (above) and PC3 (below) cells were assayed using Rho/FACS; the percentage of Rho^low^ cells are shown in the picture.

Since A549 cells grown in the 3D microenvironment matrigel acquired stem cell–like properties, we want to know whether this kind of culture can also promote the reprogramming of other cancer cells to increase the stem cell phenotype. We chose two additional types of cancer cells: MCF7 and PC3. We observed changes in the morphological features when the cells were cultured in the 3D microenvironment (Fig. 4A); the percentage of stem cell–like cells was measured in both monolayers and 3D cultures using the Rho/FACS method. As shown in Fig. 4B, the percentages of Rho^low^ cells in 2D-grown MCF7 and PC3 cells were 0.48% and 7.59%, respectively, whereas the percentages in 3D-grown MCF7 and PC3 cells were 33.7% and 73.2%, respectively. These results suggest that the 3D microenvironment can increase the stem cell phenotype of other cancer cells as well.

## DISCUSSION

Cancer cells *in vivo* interact with the ECM environment in a 3D manner, and accumulated studies have demonstrated that the tumor microenvironment plays an essential role in the carcinogenic process, metastasis and therapeutic resistance [25–27]. However, the traditional 2D monolayer cell culture ignores the important interplay between cancer cells and their local environment. Matrigel, which is rich in ECM components and mimics more closely the actual physiological environment *in vivo,* is used widely for 3D cell culture [28]. It has been demonstrated that 3D-cultured cancer cells are more radio-resistant and chemo-resistant compared with monolayer cells [13–15]. However, the mechanism that results in the difference in radio-resistance and chemo-resistance between 3D- and 2D-grown cancer cells remains largely unknown. Our current study demonstrated that cancer cells were reprogrammed and acquired a more stem cell-like phenotype when cultured in a 3D growth microenvironment compared with traditional 2D monolayer cultured cells. That was shown by the increased number of Rho^low^-staining cells. Moreover, the 3D growth microenvironment induced the cancer cells to express the reprogramming transcription factors OCT4, SOX2, NANOG and LIN28. OCT4 and SOX2 that are well known as the transcription factors that were used with c-MYC and KLF4 to generate induced pluripotent stem cells (iPSCs) [29]. However, in reprogramming human cells, it was indicated that c-MYC promoted both apoptosis and differentiation in a transcriptional activity–dependent manner [30], which suggested that this gene was not required for reprograming human cells. NANOG, as a transcription factor, is believed to play a key role in maintaining pluripotency [31], and it has been reported that OCT4 and SOX2 can bind to the promoter to activate the transcription of NANOG [32]. Increasing evidence suggests that LIN28 is a post-transcription repressor of let-7 microRNA biogenesis and an undifferentiated embryonic stem cell marker; it can also reprogram somatic cells to produce iPSCs and control the pluripotency of stem cells [33, 34]. We speculate that the upregulation of these key transcriptional factors reprogrammed the 3D-grown cells to acquire the properties of stem cells because it has been demonstrated that these four transcriptional factors, OCT4, SOX2, NANOG and LIN28, are sufficient to reprogram human somatic cells to pluripotent human cells and exhibit the essential characteristics of embryonic stem cells [35]. Furthermore, we found that the expression of miR-302a was upregulated in 3D-grown cancer cells. It has been demonstrated that the miR-302 family are not the only the key factors essential for embryonic stem cell maintenance, but also function to reprogram cancer cells into a stem cell–like pluripotent state [36]. Moreover, the expression of the miR-302 family gene has been shown to be regulated by the reprogramming factors OCT4 and SOX2 [37]. Although reprogramming that induced de-differentiation of differentiated cells to pluripotency can take place via a variety of mechanisms, accumulating evidence indicates that the stem cell activator Wnt/β-catenin signaling not only plays a critical role in maintaining pluripotency and reprogramming somatic cells to pluripotency, but also induces de-differentiation of cancer cells to acquire stem cell–like properties [38–41]. In addition, recent studies indicated that β-catenin, a key downstream component of the canonical Wnt signaling pathway, can activate OCT4, LIN28, NANOG and miR-302 expression [21–24]. Here, we found that the expression of β-catenin was upregulated in the 3D-grown cells. Thus, our results suggest that the canonical Wnt signaling pathway was activated in cancer cells when the cells were cultured in a 3D growth microenvironment and increased the expression of active β-catenin. The high level of β-catenin promoted the expression of reprogramming factors, such as OCT4, NANOG and LIN28, and upregulated the expression of miR-302a. All these reprogramming factors together reprogram cancer cells to become cancer stem-like cells. It has been demonstrated that stem-like cancer cells are radio-resistant as a result of a variety of intrinsic and extrinsic factors [42–44]. Our findings help to explain, from a new perspective, why 3D-grown cancer cells are more radio-resistant than 2D-grown cells.

It has been reported that a 3D sphere, forced by a low attachment surface or in ultra-low attachment dishes, could induce cells to express reprogramming transcription factors and acquire stem cell-like features [45–47]. However, our 3D growth model is different from the models of these researchers. In our model, cells were cultured within a 3D matrigel microenvironment. In addition, the cells acquired stem cell–like properties soon—only one day after culture in Matrigel. When cells are grown on low attachment surfaces, the cells spontaneously aggregate to form 3D spheres with cell-to-cell interactions; it becomes a 3D cell culture system that is taking advantage of the natural tendency of cells to aggregate [45]. Matrigel is a medium rich in extracellular matrix. The cultures grown in matrigel are more closely approximating the microenvironment *in vivo* and are more suitable than 2D cultures for studies on the biology of carcinogenesis.

Taken together, our results demonstrate that cancer cells were reprogrammed and acquired stem cell properties after culture in a 3D microenvironment. This provides a possible explanation as to why 3D-cultured cancer cells are more radio-resistant, compared with monolayer cancer cells. More importantly, our findings shed new light on understanding the features of the 3D growth cell model and its application in the basic research of clinical radiotherapy and medicine development.

## FUNDING

This work was supported by grants from the Hundred Talent Program of the Chinese Academy of Sciences [Y163100BR0], the 973 program from the Department of Chinese Science [2010CB834201], the National Natural Science Foundation of China [31170803], the Major Project Specialized for Infectious Diseases of the Chinese Health and Family Planning Commission [2014ZX10004002] and the National Key Scientific Instrument and Equipment Development Project of China [2012YQ03014210] to BH. Funding to pay the Open Access publication charges for this article was provided by the Major Project Specialized for infectious Diseases of the Chinese Health and Family Planning Commission [2014ZX10004002].

## References

[1] Yamada KM Pankov R Cukierman E Dimensions and dynamics in integrin function. Braz J Med Biol Res 2003;36:959–66. 12886449 10.1590/s0100-879x2003000800001

[2] Griffith LG Swartz MA Capturing complex 3D tissue physiology *in vitro*. Nat Rev Mol Cell Biol 2006;7:211–24. 16496023 10.1038/nrm1858

[3] Nelson CM Bissell MJ Of extracellular matrix, scaffolds, and signaling: tissue architecture regulates development, homeostasis, and cancer. Annu Rev Cell Dev Biol 2006;22:287–309. 16824016 10.1146/annurev.cellbio.22.010305.104315 PMC2933192

[4] Birgersdotter A Sandberg R Ernberg I Gene expression perturbation *in vitro*—a growing case for three-dimensional (3D) culture systems. Semin Cancer Biol 2005;15:405–12. 16055341 10.1016/j.semcancer.2005.06.009

[5] Kim JB Three-dimensional tissue culture models in cancer biology. Semin Cancer Biol 2005;15:365–77. 15975824 10.1016/j.semcancer.2005.05.002

[6] Rangarajan A Hong SJ Gifford A Species- and cell type-specific requirements for cellular transformation. Cancer Cell 2004;6:171–83. 15324700 10.1016/j.ccr.2004.07.009

[7] Yamada KM Cukierman E Modeling tissue morphogenesis and cancer in 3D. Cell 2007;130:601–10. 17719539 10.1016/j.cell.2007.08.006

[8] Roskelley CD Desprez PY Bissell MJ Extracellular matrix-dependent tissue-specific gene expression in mammary epithelial cells requires both physical and biochemical signal transduction. Proc Natl Acad Sci U S A 1994;91:12378–82. 7528920 10.1073/pnas.91.26.12378 PMC45441

[9] Le Beyec J Xu R Lee SY Cell shape regulates global histone acetylation in human mammary epithelial cells. Exp Cell Res 2007;313:3066–75. 17524393 10.1016/j.yexcr.2007.04.022 PMC2040058

[10] Lelievre SA Contributions of extracellular matrix signaling and tissue architecture to nuclear mechanisms and spatial organization of gene expression control. Biochim Biophys Acta 2009;1790:925–35. 19328836 10.1016/j.bbagen.2009.03.013 PMC2728154

[11] Sieh S Taubenberger AV Rizzi SC Phenotypic characterization of prostate cancer LNCaP cells cultured within a bioengineered microenvironment. PLoS One 2012;7:e40217. 22957009 10.1371/journal.pone.0040217 PMC3434144

[12] Sempere LF Gunn JR Korc M A novel 3-dimensional culture system uncovers growth stimulatory actions by TGFbeta in pancreatic cancer cells. Cancer Biol Ther 2011;12:198–207. 21613822 10.4161/cbt.12.3.15979 PMC3166497

[13] Zschenker O Streichert T Hehlgans S Genome-wide gene expression analysis in cancer cells reveals 3D growth to affect ECM and processes associated with cell adhesion but not DNA repair. PLoS One 2012;7:e34279. 22509286 10.1371/journal.pone.0034279 PMC3324525

[14] Hehlgans S Eke I Storch K Caveolin-1 mediated radioresistance of 3D grown pancreatic cancer cells. Radiother Oncol 2009;92:362–70. 19665245 10.1016/j.radonc.2009.07.004

[15] Hehlgans S Lange I Eke I 3D cell cultures of human head and neck squamous cell carcinoma cells are radiosensitized by the focal adhesion kinase inhibitor TAE226. Radiother Oncol 2009;92:371–8. 19729215 10.1016/j.radonc.2009.08.001

[16] Benton G Arnaoutova I George J Matrigel: from discovery and ECM mimicry to assays and models for cancer research. Adv Drug Deliv Rev 2014;79–80:3–18. 10.1016/j.addr.2014.06.005 24997339

[17] Lee GY Kenny PA Lee EH Three-dimensional culture models of normal and malignant breast epithelial cells. Nat Methods 2007;4:359–65. 17396127 10.1038/nmeth1015 PMC2933182

[18] Liu W-H Qian N-S Li R Replacing Hoechst33342 with rhodamine123 in isolation of cancer stem-like cells from the MHCC97 cell line. Toxicol In Vitro 2010;24:538–45. 19913610 10.1016/j.tiv.2009.11.008

[19] McKenzie JL Takenaka K Gan OI Low rhodamine 123 retention identifies long-term human hematopoietic stem cells within the Lin^−^CD34^+^CD38^−^ population. Blood 2007;109:543–5. 16990597 10.1182/blood-2006-06-030270

[20] Bertoncello I Williams B Hematopoietic stem cell characterization by Hoechst 33342 and rhodamine 123 staining. Methods Mol Biol 2004;263:181–200. 14976367 10.1385/1-59259-773-4:181

[21] Cai W-Y Wei T-Z Luo Q-C The Wnt–β-catenin pathway represses let-7 microRNA expression through transactivation of Lin28 to augment breast cancer stem cell expansion. J Cell Sci 2013;126:2877–89. 23613467 10.1242/jcs.123810

[22] Teng Y Wang X Wang Y Wnt/β-catenin signaling regulates cancer stem cells in lung cancer A549 cells. Biochem Biophys Res Commun 2010;392:373–9. 20074550 10.1016/j.bbrc.2010.01.028

[23] Takao Y Yokota T Koide H β-catenin up-regulates Nanog expression through interaction with Oct-3/4 in embryonic stem cells. Biochem Biophys Res Commun 2007;353:699–705. 17196549 10.1016/j.bbrc.2006.12.072

[24] Brautigam C Raggioli A Winter J The Wnt/β-catenin pathway regulates the expression of the miR-302 cluster in mouse ESCs and P19 cells. PLoS One 2013;8:e75315. 24040406 10.1371/journal.pone.0075315 PMC3769259

[25] He H Tian D Guo J DNA damage response in peritumoral regions of oesophageal cancer microenvironment. Carcinogenesis 2013;34:139–45. 23027622 10.1093/carcin/bgs301

[26] Sounni NE Noel A Targeting the tumor microenvironment for cancer therapy. Clin Chem 2013;59:85–93. 23193058 10.1373/clinchem.2012.185363

[27] Sung SY Hsieh CL Wu D Tumor microenvironment promotes cancer progression, metastasis, and therapeutic resistance. Curr Probl Cancer 2007;31:36–100. 17362788 10.1016/j.currproblcancer.2006.12.002

[28] Herrmann D Conway JR Vennin C Three-dimensional cancer models mimic cell–matrix interactions in the tumour microenvironment. Carcinogenesis 2014;35:1671–9. 24903340 10.1093/carcin/bgu108

[29] Takahashi K Yamanaka S Induction of pluripotent stem cells from mouse embryonic and adult fibroblast cultures by defined factors. Cell 2006;126:663–76. 16904174 10.1016/j.cell.2006.07.024

[30] Sumi T Tsuneyoshi N Nakatsuji N Apoptosis and differentiation of human embryonic stem cells induced by sustained activation of c-Myc. Oncogene 2007;26:5564–76. 17369859 10.1038/sj.onc.1210353

[31] Chambers I Colby D Robertson M Functional expression cloning of Nanog, a pluripotency sustaining factor in embryonic stem cells. Cell 2003;113:643–55. 12787505 10.1016/s0092-8674(03)00392-1

[32] Rodda DJ Chew JL Lim LH Transcriptional regulation of nanog by OCT4 and SOX2. J Biol Chem 2005;280:24731–7. 15860457 10.1074/jbc.M502573200

[33] Darr H Benvenisty N Genetic analysis of the role of the reprogramming gene *LIN-28* in human embryonic stem cells. Stem Cells 2009;27:352–62. 19038789 10.1634/stemcells.2008-0720

[34] Qiu C Ma Y Wang J Lin28-mediated post-transcriptional regulation of Oct4 expression in human embryonic stem cells. Nucleic Acids Res 2010;38:1240–8. 19966271 10.1093/nar/gkp1071 PMC2831306

[35] Yu J Vodyanik MA Smuga-Otto K Induced pluripotent stem cell lines derived from human somatic cells. Science 2007;318:1917–20. 18029452 10.1126/science.1151526

[36] Lin S-L Chang D-C Chang-Lin S Mir-302 reprograms human skin cancer cells into a pluripotent ES-cell-like state. RNA 2008;14:2115–24. 18755840 10.1261/rna.1162708 PMC2553732

[37] Card DA Hebbar PB Li L Oct4/Sox2-regulated miR-302 targets cyclin D1 in human embryonic stem cells. Mol Cell Biol 2008;28:6426–38. 18710938 10.1128/MCB.00359-08 PMC2577422

[38] Miki T Yasuda SY Kahn M Wnt/β-catenin signaling in embryonic stem cell self-renewal and somatic cell reprogramming. Stem Cell Rev 2011;7:836–46. 21603945 10.1007/s12015-011-9275-1

[39] Lluis F Pedone E Pepe S Periodic activation of Wnt/beta-catenin signaling enhances somatic cell reprogramming mediated by cell fusion. Cell Stem Cell 2008;3:493–507. 18983965 10.1016/j.stem.2008.08.017

[40] Cheng Y Cheung AK Ko JM Physiological β-catenin signaling controls self-renewal networks and generation of stem-like cells from nasopharyngeal carcinoma. BMC Cell Biol 2013;14:44. 24073846 10.1186/1471-2121-14-44 PMC3819748

[41] Hsieh IS Chang KC Tsai YT MicroRNA-320 suppresses the stem cell-like characteristics of prostate cancer cells by downregulating the Wnt/β-catenin signaling pathway. Carcinogenesis 2013;34:530–8. 23188675 10.1093/carcin/bgs371

[42] Hittelman WN Liao Y Wang L Are cancer stem cells radioresistant? Future Oncol 2010;6:1563–76. 21062156 10.2217/fon.10.121 PMC3059151

[43] Carruthers R Ahmed SU Strathdee K Abrogation of radioresistance in glioblastoma stem-like cells by inhibition of ATM kinase. Mol Oncol 2015;9:192–203. 25205037 10.1016/j.molonc.2014.08.003 PMC5528679

[44] Zhang X Komaki R Wang L Treatment of radioresistant stem-like esophageal cancer cells by an apoptotic gene-armed, telomerase-specific oncolytic adenovirus. Clin Cancer Res 2008;14:2813–23. 18451249 10.1158/1078-0432.CCR-07-1528 PMC2387204

[45] Su G Zhao Y Wei J The effect of forced growth of cells into 3D spheres using low attachment surfaces on the acquisition of stemness properties. Biomaterials 2013;34:3215–22. 23439133 10.1016/j.biomaterials.2013.01.044

[46] Debeb BG Zhang X Krishnamurthy S Characterizing cancer cells with cancer stem cell-like features in 293 T human embryonic kidney cells. Mol Cancer 2010;9:180. 20615238 10.1186/1476-4598-9-180 PMC2915978

[47] Su G Zhao Y Wei J Direct conversion of fibroblasts into neural progenitor-like cells by forced growth into 3D spheres on low attachment surfaces. Biomaterials 2013;34:5897–906. 23680365 10.1016/j.biomaterials.2013.04.040

